# Placebo Effects on Visual Food Cue Reactivity: An Eye-Tracking Investigation

**DOI:** 10.3389/fpsyt.2019.00525

**Published:** 2019-07-24

**Authors:** Jonas Potthoff, Nina Jurinec, Anne Schienle

**Affiliations:** Department of Clinical Psychology, University of Graz, Graz, Austria

**Keywords:** visual food cue reactivity, placebo, eye-tracking, appetite, wanting, liking

## Abstract

**Background:** Enhanced visual food cue reactivity has been associated with overeating and weight gain. Due to the increasing prevalence of high-fat food images that we are constantly exposed to in both the real and the virtual world, methods that are able to reduce the reactivity to these types of cues are urgently needed. This eye-tracking study investigated whether food cue reactivity, especially toward high-caloric food, can be reduced with a placebo intervention.

**Method:** Fifty-two women [mean body mass index (BMI) = 23.5] were presented with pictures depicting combinations of food (high-caloric, low-caloric) and non-food items, which were shown once with and once without a placebo in a repeated-measures design. The placebo was a pill introduced as a medication targeting peptide YY that is able to reduce appetite specifically for high-caloric food. Gaze data (dwell time, fixations) and self-reported appetite were assessed during the two eye-tracking sessions (with/without placebo).

**Results:** The placebo reduced general appetite as well as specific appetite for the depicted food items. Additionally, the placebo decreased the percentage of fixations and dwell time on the food images. The placebo was not able to specifically change visual food cue reactivity to high-caloric stimuli but reduced responses to both high-caloric and low-caloric food. Reported appetite reduction and weight concerns were positively associated with the placebo-related decrease in visual attention for food.

**Conclusions:** The placebo was able to reduce visual food cue reactivity. This finding demonstrates that placebos are able to alter early visual–attentional processes.

## Introduction

Food is a primary reinforcer that attracts automatic attention. From an evolutionary perspective, this mechanism enhances the efficient detection of food sources in the environment, which, in turn, enables adequate food intake and thus survival [e.g., Ref. (1)].

Neurobiological studies with methods such as electroencephalography, functional magnetic resonance imaging, and eye-tracking have revealed evidence that the human attentional system is tuned to identify food targets very quickly and to differentiate them from non-food items [e.g., Refs. (2–4)]. In addition, high-caloric food captures more automatic attention than low-caloric food (4, 5). This attention bias seems to be more pronounced in overweight participants. Castellanos et al. (4) recorded eye movements for picture pairs with food (high-caloric, low-caloric) and non-food items during both a fasted and a fed condition, in normal-weight and obese women. In the fasted condition, both groups demonstrated longer fixation duration for food compared to non-food images. This visual bias was especially pronounced for high-caloric food. In the fed condition, obese individuals maintained increased attention towards food images. Additionally, they directed their first fixation toward food images more often than normal-weight individuals did. Similar findings were reported by Werthmann et al. (6). Overweight women directed more initial attention (first fixations) toward images with high-fat food than normal-weight women. In a more recent study by Doolan et al. (5), normal weight and overweight adults (men and women) viewed high-caloric, low-caloric food and control images, during both a fasted and fed condition. Participants directed greater visual attention towards high-caloric food images. This response was most pronounced in overweight men.

The response bias for high-caloric food described above has been advantageous in earlier times when humans were still hunter-gatherers. However, in the present time, it has become problematic in Western societies due to the food surplus, compounded with the constant exposure to visual food cues, both in the virtual world (e.g., cookery shows on TV, food blogs) and in the real world (e.g., in supermarkets, restaurants) (7). This type of stimulation elicits appetite and the urge to consume the displayed food items [e.g., Refs. (8, 9)]. Since these food cues are so prevalent, it is not surprising that individual food cue reactivity can predict overeating, subsequent weight gain, and risk of obesity [see meta-analysis by Ref. (10)].

Modifying visual food cue reactivity is therefore a promising method for altering overeating habits. According to Boswell and Kober (10), food cue reactivity involves conditioned responses to stimuli that signal the presence of food (e.g., visual, olfactory cues), including physiological reactivity and craving. To change food cue reactivity, there are different behavioral strategies available. For instance, situation selection (where a person chooses to go into or avoid certain situations) and situation modification (where a person actively changes a situation, such as preference of diet products) are such strategies. Furthermore, cognitive reappraisal can be carried out. This refers to interpreting a situation in a way that alters its emotional impact (11). For example, one might focus on the negative consequences of food consumption, such as weight gain, or tell oneself that although a food item looks appetizing, it is not healthy. Such cognitive reappraisal strategies can reduce food cue reactivity [e.g., Refs. (12–14)]. However, all of the aforementioned strategies involve explicit cognitive processes that are effortful. These effortful inhibitory processes are generally challenging, but even more so for those who exhibit a tendency to overeat [e.g., Refs. (2, 15, 16)].

Due to the challenges involved in reducing food cue reactivity with explicit cognitive strategies, alternative (implicit) strategies should be considered. One such strategy is placebo treatment. Placebos are substances or treatments that are physically or pharmacologically inert. These types of treatments are offered to a recipient with the verbal suggestion that somatic and/or affective processes will change in a specific way (17). The most studied placebo effect is “placebo analgesia” (a reduction in pain that can be attributed to a sham treatment). Emerging neuroscience evidence implicates that multiple brain systems and neurochemical mediators are involved in placebo analgesia. Studies using the electroencephalogram have shown that placebo treatments are able to reduce amplitudes of event-related potentials in response to painful stimuli [e.g., Ref. (18)]. These changes occur already ∼100–200 ms after the onset of noxious stimulation, indicating early attentional and perceptual effects of placebos. However, placebo analgesia is also associated with autonomic and endocrine changes that occur much later [in the time frame of minutes and hours; for a review, see Ref. (17)]. A placebo therefore has several effects depending on the effector and time window investigated.

Studies in the area of appetite regulation have also consistently demonstrated placebo effects. Placebo-controlled clinical trials of appetite suppressants [e.g., Ref. (19] and placebo studies with healthy participants [e.g., Refs. (20, 21)] or with patients suffering from eating disorders [e.g., Ref. (22)] have all identified appetite-changing effects of sham treatments. For example, Hoffmann et al. (21) found that a satiety-enhancing placebo reduced reported appetite. An appetite-enhancing placebo did not alter subjective levels of hunger, but increased plasma levels of the “hunger hormone” ghrelin in female participants.

To the best of our knowledge, placebo-induced changes in food cue reactivity and appetite have not been studied with eye-tracking so far. Such studies are important in order to find out if appetite-reducing placebos are able to affect early attentional–perceptual processes. The design of the current study was based on an experiment by Schienle et al. (23) during which the subjects passively viewed picture pairs (disgust pictures, neutral pictures) once with and once without a “disgust placebo” (inert pill administered with the verbal suggestion that it would reduce disgust symptoms). The placebo lowered reported revulsion and enhanced the fixation duration for disgusting pictures. The authors suggested that this change while on the placebo reflected a greater willingness of the participants to view these (previously avoided) stimuli.

The present placebo investigation administered picture pairs that depicted food (high-caloric, low-caloric) and non-food items. The experiment had a repeated-measures design with two counter-balanced sessions: the female participants viewed the pictures once with and once without the placebo. The placebo was introduced as a medication that targets peptide YY (a peptide released from cells in response to eating and satiety), which is able to reduce appetite, especially for high-caloric food. It was expected that the placebo would reduce the visual preference for high-caloric food cues (as indexed by reduced percentages of fixations, dwell time, and reduced initial gaze direction), as well as the reported appetite for high-caloric food [e.g., Ref. (24)]. Furthermore, a regression approach was used in order to analyze whether reported concerns about weight and eating as well as body mass index (BMI) would be associated with placebo-related effects on eye movements and appetite. This was done in order to investigate if overweight women who would like to lose weight might profit from this type of placebo intervention.

## Methods

### Sample

Fifty-two women (mean age: 26.4 years, SD = 8.7) with a mean BMI of 23.5 (SD = 3.7) took part in this experiment. Of the participants, nine were overweight (BMI = 25–30) and three were obese (BMI > 30) (**Table 1**). Sixty-nine percent of the participants were university students; the remaining subjects were white-collar workers. They had normal or corrected-to-normal vision and did not report any somatic or mental disorders and no intake of medication. Participants were recruited for a study of an appetite-reducing medication (“propionate”) *via* email lists and postings at the university campus. Written informed consent was obtained from all participants. The study was approved by the ethics committee of the university and was conducted in accordance with the Declaration of Helsinki.

**Table 1:** Sample characteristics.

Measure	Mean (SD)	Minimum	Maximum
Age (years)	26.4 (8.7)	18	52
BMI (kg/m^2^)	23.5 (3.7)	17.8	35.0
EDE-Q Eating Concern	1.3 (1.2)	0.0	4.6
EDE-Q Weight Concern	2.4 (1.5)	0.0	5.6
Placebo effectiveness	3.3 (1.9)	0.0	6.0

Placebo effectiveness: from 1 = “No change in appetite at all” to 7 = “Highest effect imaginable.” BMI, body mass index; EDE-Q, Eating Disorder Examination-Questionnaire.

### Stimuli

The stimulus material comprised 60 images from the categories “low-caloric food” (e.g., fruits), “high-caloric food” (e.g., cream cakes), and “non-food” (e.g., office supplies). All images were taken from a validated set by Blechert et al. (25) and had a size of 600 × 450 pixels. The images of the three categories (high-caloric, low-caloric, and non-food) did not differ in their RGB values [R: *F*(2,57) = .952, *p* = .392, η^2^_p_ = .032; G: *F*(2,57) = .789, *p* = .459, η^2^_p_ = .027; B: *F*(2,57) = 1.729, *p* < .187, η^2^_p_ = .057] and their object size (number of pixels that are not the background) [*F*(2,57) = .033, *p* = .968, η^2^_p_ = .001].

**Figure 1:**
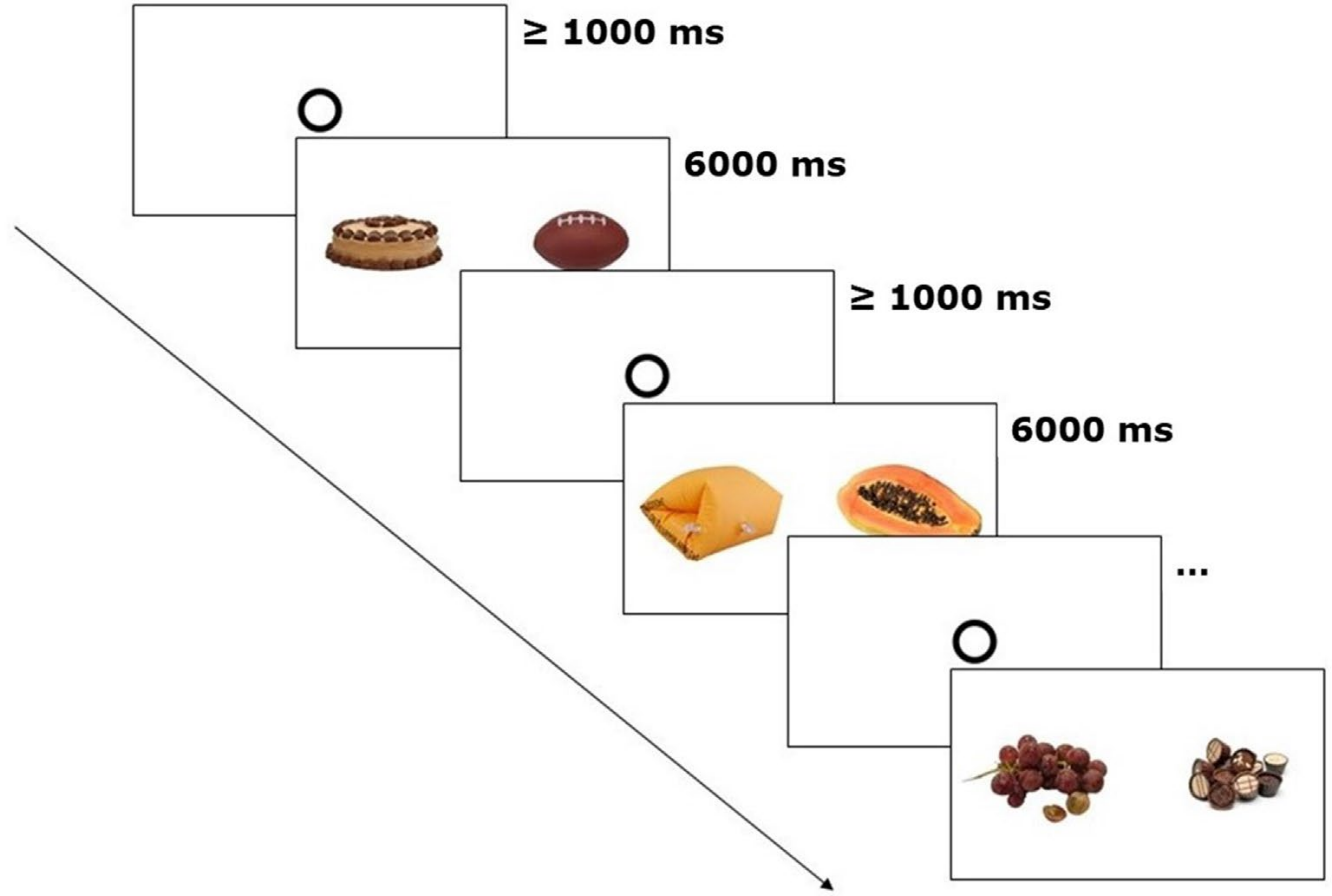
Eye-tracking paradigm. After every 20 trials, general appetite was rated. The depicted trials show one image pair of each category (high-caloric + non-food, low-caloric + non-food, high-caloric + low-caloric). Fixation disks had to be looked at for at least 1,000 ms in order to start the next trial.

The stimuli were presented as image pairs side by side on a white background on the computer screen (see **Figure 1**). Three types of image pairs were created: high-caloric + low-caloric food (*n* = 10), high-caloric food + non-food (*n* = 10), and low-caloric + non-food (*n* = 10). Each image pair was shown twice during the experiment (60 trials in total). The second time an image pair was presented, the arrangement (which image was on the left or right side of the screen) was mirrored. The trial order was randomized. The eye-tracking experiment with the picture presentation lasted approximately 8 min.

### Procedure

All participants answered demographic questions and two subscales of the Eating Disorder Examination-Questionnaire (EDE-Q) by Hilbert et al. (26) *via* an online survey (weight concern, eating concern). The questions are concerned with the past 4 weeks and are answered on seven-point scales (0 = not at all; 6 = very much). Typical items of the weight concern scale are: “How dissatisfied are you with your weight?” “Did you have the strong desire to lose weight?”; eating concern: “Were you afraid to lose control over your eating?” Cronbach’s alphas in the present sample were α = .82 (weight concern) and α = .83 (eating concern).

Then, 52 participants were invited to the eye-tracking experiment [the sample size had been determined based on a previous eye-tracking study with a comparable design; see Ref. (23)]. The experiment had a repeated-measures design and consisted of two sessions (with and without placebo), which were conducted approximately 1 week apart. The sequence of the two sessions (Placebo first vs. No Placebo first) was counterbalanced (26:26) across participants. Both sessions were conducted during the same time of the day after a 3-h fast. At the beginning of each session, the participants rated their general appetite on a seven-point scale (1: “I have no appetite at all;” 7: “I have an extreme urge to eat something right now”). This rating was repeated after 20, 40, and 60 trials.

The participants were asked to look at the images as if they were watching TV. Similar free-exploration instructions have been used before to study attentional biases in visual food cue perception (27, 28). Each image pair was shown for 6 s. Prior to each trial, a circle in the center of the screen had to be fixated for 1 s. Subsequently, the free exploration trial started, the circle disappeared, and the image pair was shown (**Figure 1**).

At the end of the each of the two sessions, 15 of the presented images (5 low-caloric food items, 5 high-caloric food items, 5 non-food items) were shown again in random order. The 15 of the 60 stimuli pictures were chosen in order to cover a wide variety of different food items (e.g., cake, chocolate, fruits) but not to prolong the study. We presented only 15 images to avoid fatigue, effort, and boredom associated with repeated rating. The participants were asked to rate these food items with regard to their specific appetite/wanting (“How much would you like to taste this food right now?” 1: “not at all”, 7: “very much”) and liking (“How much do you like this food in general?” 1: “not at all”, 7: “very much”).

**Figure 2:**
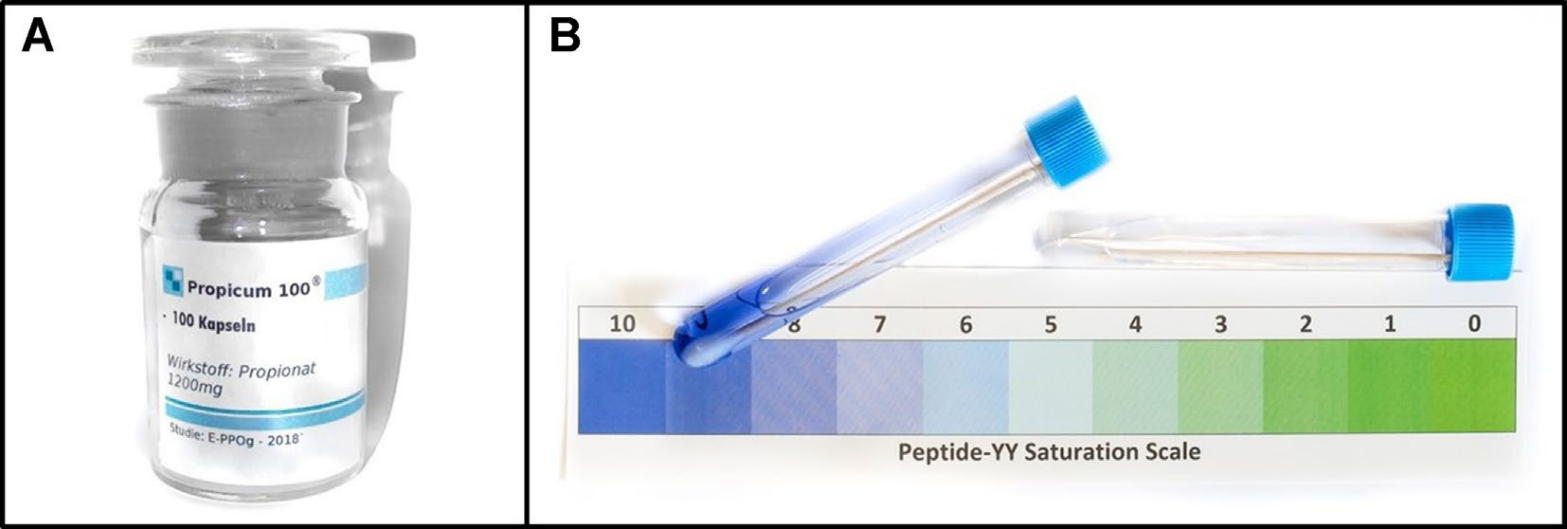
Placebo material. Left **(A)**: placebo pill container; right **(B)**: sham saliva test.

In the placebo condition, the participants received a placebo pill (a 1-cm-long silica-filled capsule) prior to the picture presentation with the following verbal suggestion: “This pill contains propionate. The appetite-reducing effect of propionate, especially for high-calorie food, has repeatedly been confirmed in previous studies. The decrease in appetite is triggered by the release of the hormones peptide YY and glucagon-like peptide 1 (GLP-1). The effect will be noticeable approximately 15 minutes after intake.” During this waiting time, the participants read an abstract of a scientific article and a newspaper article about propionate describing the positive effects of this medication. Subsequently, a saliva sample was taken from each participant and the experimenter pretended to conduct a test on the peptide YY level. The test fluid changed in color from colorless to blue (for all participants). It was explained that this would indicate a high peptide YY level (**Figure 2**). After the saliva test, the participants rated the effectiveness of propionate on a seven-point scale (7 = “extremely effective”; 1 = “not effective”).

### Eye Movement Recording and Analysis

We recorded two-dimensional eye movements using an SMI RED250 mobile eye-tracker with a sampling rate of 250 Hz. To minimize head movements, a chin rest was used. We calibrated both eyes and analyzed data from the eye that produced the better spatial resolution, which was better than 0.35° visual angle. Stimuli were presented on a white background on a 24-in. screen with a resolution of 1920 × 1080 pixels. The viewing distance was 60 cm, resulting in a size of 15.6° × 11.7° viewing angle for the shown images. The experiment was controlled *via* the SMI Experiment Center (Version 3.6.53). For event detection, standard thresholds of the SMI BeGaze Software (Version 3.6.52) for high-speed eye-tracking data (sampling rate > 200 Hz) were used: The standard velocity threshold for saccade detection was 40°/s. In line with this velocity-based threshold [see Ref. (29)], fixations were defined by an absence of saccades and blinks (defined as moments without registered gaze positions) that lasted at least 50 ms. Data were exported using SMI BeGaze and customized Python scripts. Within BeGaze, we defined the food and non-food images as areas of interest (AOI). We conducted gaze data analysis exclusively for the two AOIs of each trial.

We computed the percentage of fixations and dwell time that was spent on the food image (either high-caloric or low-caloric). For image pairs containing high-caloric and low-caloric food, these percentages were computed for the high-caloric image (for example, a value of 70% indicates that from the total number of fixations/dwell time, 70% were directed to the high-caloric food and 30% to the low-caloric food). Furthermore, the location of the first fixation was determined and used to compute the percentage of trials in which the first fixation was on the food image. For descriptive data (number of fixations and dwell time on each AOI), see **Table 2**.

**Table 2:** Descriptive statistics (means, standard deviations) for the gaze parameters during the Placebo and No Placebo condition.

Image pair	Image	Mean number of fixations (SD)		Mean dwell time in ms (SD)	
		Placebo	No Placebo	Placebo	No Placebo
HCLC	HC	5.6 (1.8)	6.2 (1.6)	2,382.5 (722.1)	2,786.2 (779.8)
	LC	6.2 (1.5)	5.5 (1.3)	2,756.0 (689.1)	2,436.7 (696.0)
HCNF	HC	6.0 (1.8)	6.5 (1.7)	2,767.0 (895.0)	3,178.6 (909.6)
	NF	5.7 (1.9)	4.8 (1.6)	2,414.8 (800.7)	2,018.6 (781.1)
LCNF	LC	5.7 (1.5)	6.2 (1.3)	2,894.4 (858.3)	3,221.9 (749.0)
	NF	5.3 (1.8)	4.8 (1.6)	2,230.8 (708.8)	2,007.6 (667.4)

HCLC, high-caloric and low-caloric; HCNF, high-caloric and non-food; LCNF, low-caloric and non-food.

### Statistical Analyses

In order to investigate placebo effects on general appetite, an analysis of variance (ANOVA) for repeated measures was computed with the within-subject factors Treatment (Placebo, No Placebo) and Time of Measurement (at the beginning of the session, after 20 trials, after 40 trials, after 60 trials of image presentation).

To evaluate the effect of the placebo treatment on the wanting/liking of the food depicted in the images, ANOVAs for repeated measures were computed with the within-subject factors Treatment (Placebo, No Placebo) and Image Category (high-caloric, low-caloric food) (the non-food items elicited no appetite and were therefore excluded from the analysis).

ANOVAs for repeated measures were performed with the within-subject factors Image Pair Category (high-caloric + non-food, low-caloric + non-food, high-caloric + low-caloric) and Treatment (Placebo, No Placebo) for percentage of fixations, first fixations, and dwell time.

If sphericity was violated (Mauchly’s Test of Sphericity), Greenhouse–Geisser correction was applied. We report the effect size as η^2^_p_ (partial eta squared) and Bonferroni adjusted *p* values. *p* values smaller than .05 were considered to be statistically significant.

Prior to the statistical analyses, we investigated a possible effect of the sequence of sessions (session with placebo first vs. session without placebo first). The calculated ANOVAs for general appetite, wanting, liking, fixations, dwell time, and first fixations revealed no significant interaction effects (all *p* > .10). Therefore, the sequence factor was not included in the ANOVAs.

Furthermore, we calculated three multiple linear regression analyses (enter method) to estimate the relationship between placebo-related changes of fixations on food, dwell time, and appetite (dependent variables) and the predictors eating concern, weight concern (EDE-Q scores), and BMI. In order to reveal possible associations between placebo-induced changes in appetite and percentage of dwell time on food images as well as percentage of fixations on food images, two exploratory Pearson correlations were calculated.

## Results

### Self-Report

*EDE-Q*: The participants obtained the following scores on the selected EDE-Q subscales: *M* = 1.3 (*SD* = 1.2) for eating concern and *M* = 2.4 (*SD* = 1.5) for weight concern. Both eating concern [*t*(51) = 3.1, *p* = .003] and weight concern [*t*(51) = 3.4, *p* = .001] were elevated compared to the healthy norm sample (26).

*Placebo effectiveness:* The rated effectiveness of the placebo was, on average, *M* = 3.3 (*SD* = 1.9). A higher rating of placebo effectiveness was associated with a greater appetite reduction during the presentation of the food images (appetite rated before minus after placebo administration; *r* = −.36, *p* < .01).

**Figure 3:**
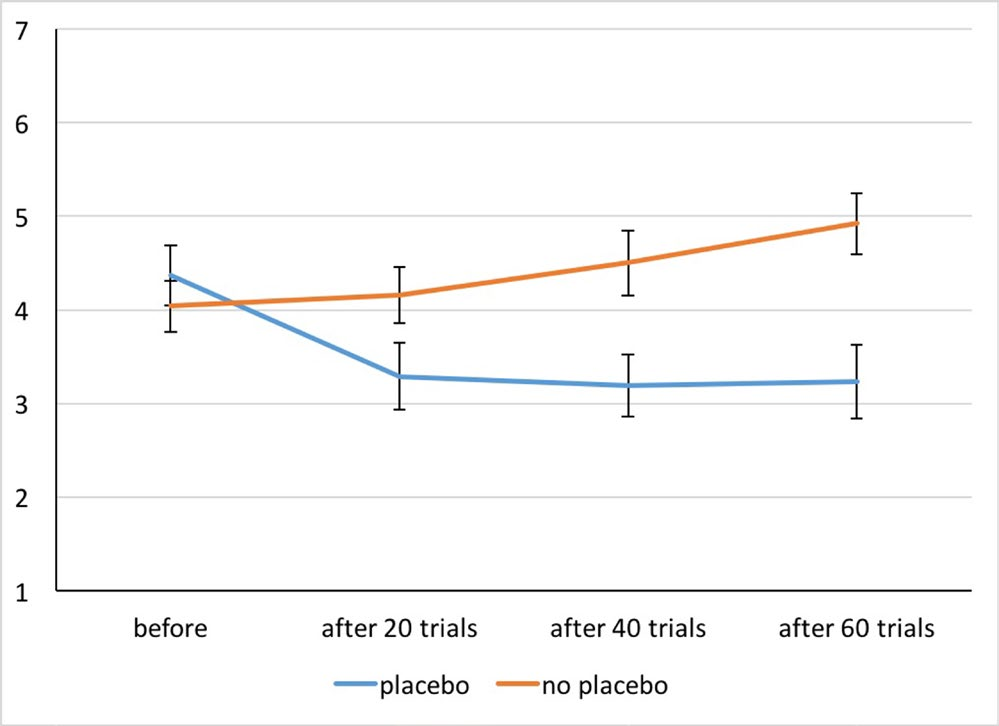
Appetite ratings for both conditions before and after 20, 40, and 60 trials of picture presentation. Whiskers indicate Cousineau–Morey confidence intervals (29).

*General appetite ratings*: The performed ANOVA revealed significant main effects of Treatment [*F*(1,51) = 12.84, *p* = .001, η^2^_p_ = .20] and Time [*F*(2.34,119.25) = 9.49, *p* < .001, η^2^_p_ = .16] and the Interaction [*F*(1.75,89.39) = 36.53, *p* < .001, η^2^_p_ = .42]. (**Figure 3**). *Post hoc* *t* tests indicated that in the No Placebo condition, reported appetite increased from the first assessment (beginning of session) to the third and fourth assessment (after 40 and 60 trials of picture presentation; both *p* < .002). In the Placebo condition, the reported appetite was lower after 20, 40, and 60 trials of picture presentation compared to the initial value prior to placebo administration (all *p* < .001). The comparison of the Placebo and No Placebo condition showed that appetite ratings did not differ at the beginning of the session (*p* = .15) but for all other assessments (after 20, 40 and 60 trials of picture presentation, all *p* < .003). All *post hoc* tests were significant after Bonferroni correction.

*Wanting and liking of presented food images*: The ANOVA for wanting revealed a main effect of Treatment [*F*(1,51) = 30.78, *p* < .001, η^2^_p_ = .38] with lower values in the Placebo condition (*M* = 3.0, *SD* = 1.3) relative to the No Placebo condition (*M* = 4.2, *SD* = 1.2). The effect of Image Category [*F*(1,51) = 34.83, *p* < .001, η^2^_p_ = .41] was also significant with higher ratings for low-caloric (*M* = 4.1, *SD* = 1.3) vs. high-caloric food (*M* = 3.1, SD = 1.0). The interaction Treatment × Image Category did not reach statistical significance [*F*(1,51) = .15, *p* = .70, η^2^_p_ = .003].

For food liking, the main effect of Image Category was statistically significant [*F*(1,51) = 44.16, *p* < .001, η^2^_p_ = .46] with higher ratings for low-caloric food (low-caloric: *M* = 5.3, *SD* = 1.1; high-caloric: *M* = 3.9, *SD* = 1.0). The main effect of Treatment [*F*(1,51) = 3.17, *p* = .08, η^2^_p_ = .06] and the interaction Treatment × Image Category did not reach significance [*F*(1,51) = .43, *p* = .52, η^2^_p_ = .008].

### Eye Movements

*Fixations*: The ANOVA revealed a significant main effect of Treatment [*F*(1,51) = 9.18, *p* = .004, η^2^_p_ = .15] with a reduced percentage of fixations on food pictures during placebo treatment (Placebo: *M* = 51.4%, *SD* = 10.3%; No Placebo: *M* = 56.9%, *SD* = 10.3%). The main effect of Image Pair Category [*F*(1.48,75.60) = 1.05, *p* = .34, η^2^_p_ = .02] and the interaction Treatment × Image Pair Category did not reach statistical significance [*F*(1.72,87.84) = 1.08, *p* = .34, η^2^_p_ = .02].

**Figure 4:**
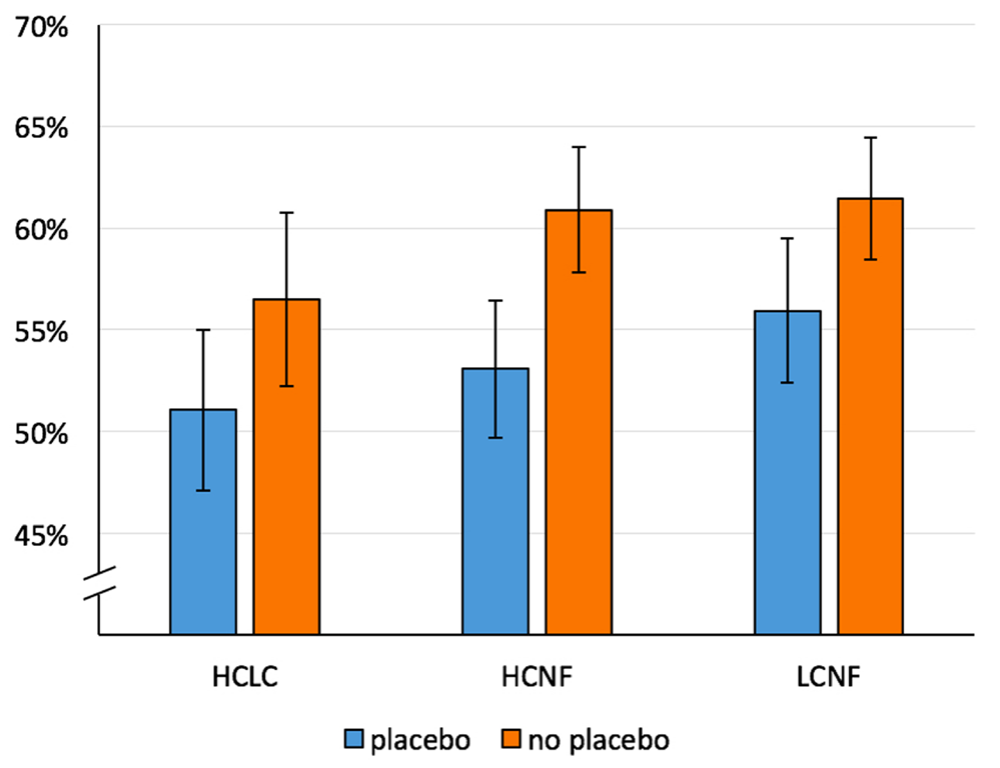
Mean percentage of dwell time on food for both conditions (Placebo, No Placebo) and three image pair conditions: HCLC (high-caloric food paired with low-caloric food; percentage of dwell time on high-caloric food), HCNF (high-caloric food paired with non-food), and LCNF (low-caloric food paired with non-food). Whiskers indicate Cousineau–Morey confidence intervals (30).

*Dwell time*: The main effect of Treatment [*F*(1,51) = 7.94, *p* = .007, η^2^_p_ = .14] was significant and indicated a placebo-related reduction in percentage of dwell time on food pictures (see **Figure 4**). The main effect of Image Pair Category was also significant [*F*(1.39,71.06) = 4.01, *p* = .04, η^2^_p_ = .07], but the computed *post hoc* *t* tests were not significant after Bonferroni correction. The interaction effect Treatment × Image Pair Category did not reach statistical significance [*F*(1.54,78.37) = .91, *p* = .38, η^2^_p_ = .02].

*First fixations*: The main effect of Image Pair Category was significant [*F*(2,102) = 19.74, *p* < .001, η^2^_p_ = .28]. First fixations were directed more often on high-caloric food (*M* = 52.6%, *SD* = 6.2%) than on low-caloric food (*M* = 47.7%, *SD* = 6.6%) when presented simultaneously with non-food items [*t*(51) = 3.65, *p* = .001]. The main effect of Treatment [*F*(1,51) = .49, *p* = .49, η^2^_p_ = .009] as well as the interaction Treatment × Image Pair Category did not reach statistical significance [*F*(2,102) = .61, *p* = .55, η^2^_p_ = .01].

*Exploratory correlation analyses*: A decrease in fixations on food presented in image pairs with non-food (percentage of fixations on food with placebo minus percentage of fixations on food without placebo) was associated with reduced appetite (mean appetite during the eye-tracking paradigm within placebo session minus mean appetite during the eye-tracking paradigm during control session) (*r* = .424, *n* = 52, *p* = .002). Furthermore, we found a significant correlation (*r* = .444, *n* = 52, *p* = .001) between appetite reduction and dwell time on food.

*Regression analyses*: For placebo-related fixation changes (percentage of fixations on food without placebo minus percentage of fixations on food with placebo), a significant equation with an adjusted *R*² of .11 was found [*F*(3,48) = 3.17, *p* = .03]. Weight concern was a significant positive predictor (**Table 3**). More pronounced weight concerns were associated with greater placebo-related reduction of food fixation. For changes in dwell time (percentage of dwell time on food without placebo minus percentage of dwell time on food with placebo), a significant regression equation was found [*F*(3,48) = 3.65, *p* = .02] with an adjusted *R*² of .14. Weight concern was a significant positive predictor of change in dwell time percentage (see **Table 3**). For change in appetite (appetite before minus after placebo treatment), no significant model was found.

**Table 3:** Association between changes in fixation percentage, dwell percentage, and appetite (dependent variables) and EDE-Q eating concern, EDE-Q weight concern, and BMI (predictors).

	*B* (SE)	Stand. *B*	95% CI for *B*	*p*
**Fixation %**				
Weight concern	.052 (.019)	.619	[−.090;−.015]	.007
Eating concern	.030 (.022)	.288	[−.014;.074]	.177
BMI	.003 (.005)	.098	[−.007;.104]	.499
**Dwell %**				
Weight concern	.070 (.023)	.670	[−.116;−.024]	.003
Eating concern	.042 (.027)	.328	[−.011;.096]	.120
BMI	.006 (.006)	.129	[−.007;.018]	.371
**Appetite**				
Weight concern	.244 (.189)	.301	[−.135;.624]	.202
Eating concern	.116 (.223)	.115	[−.565;.333]	.606
BMI	.023 (.051)	.069	[−.080;.126]	.655

## Discussion

Given the increasing prevalence of high-fat food images that surround us in both the real and virtual world, and dysfunctional eating behavior associated with this, it is important to find ways to reduce visual attention towards high-energy food. In the current eye-tracking experiment, participants were presented with images of food (high-caloric/low-caloric) and non-food items. These images were shown once in combination with a placebo (an inert pill introduced as a medication that is able to specifically reduce appetite for high-caloric food) and once without the placebo.

The repeated presentation of visual food cues increased the reported appetite of the participants. In the No Placebo condition, the general appetite (desire to eat something) gradually increased across the trials. The placebo stopped this increase. Even during the first assessment of appetite during the eye-tracking experiment (after having viewed the first 20 picture pairs), the women in this condition experienced appetite reduction due to the placebo treatment. This reduced appetite continued to be present during the course of the entire experiment. In line with the general reduction of appetite, participants reported that their specific appetite for the depicted food items (“food wanting”) was also reduced by the placebo. Thus, the placebo was able to reduce the desire to eat. The changes in self-report were in line with the eye-tracking data. The placebo pill reduced the percentage of fixations and the dwell time on food pictures. While under the placebo, the participants looked more often at the non-food items relative to the food (high-caloric and low-caloric).

The current study demonstrated a placebo effect on attentional processes that became apparent after a few minutes. This finding is in line with previous neurobiological studies, which also detected placebo-related changes in attentional networks of the brain in the range of milliseconds and seconds [e.g., Refs. (18, 23, 31, 32)]. In the mentioned EEG experiments (18, 32), a placebo was able to alter event-related components that reflect motivated attention (the characteristic of emotionally relevant stimuli to capture automatic attention). The studies with functional magnetic resonance imaging (30) showed that the placebo was able to change activation in primary and secondary visual cortex areas during the processing of affective pictures. Altogether, these results indicate that initial placebo effects rely on the modulation of sensory–attentional processes.

Furthermore, this modulation of attention could be predicted based on reported weight concerns of the participants. As shown on the regression analyses, dissatisfaction with one’s own weight and the desire to lose weight (EDE-Q scale weight concern) were positively associated with placebo responsiveness; this was true for both gaze indicators (fixations and dwell time on food pictures). Miller et al. (33) have investigated the placebo effect in the context of illness and interpersonal healing. They argue that placebos predominantly operate by producing symptomatic relief of illness (e.g., pain, anxiety). This concept implies that some degree of impairment (suffering) must be present for a placebo to be able to work and to be effective. In the current experiment, the placebo was particularly beneficial for those women who perceived their own weight as problematic and who hoped for an appetite reduction. The BMI was not able to predict the gaze indicators of FCR. Therefore, our findings suggest that not the weight status itself (being overweight) but the subjective perception of one’s own weight is a crucial predictor for the effectiveness of the placebo treatment.

We need to mention the following limitations of the current study. We analyzed the effect of a placebo on responses toward food cues in a female sample of university students (69%), who on average reported elevated eating and weight concerns and therefore were motivated to participate in the “propionate” study. Future studies should include clinical interviews for reliable diagnoses of possible eating disorders. Due to the self-selection of the participants, our findings cannot be generalized to other populations. Further, the reported food wanting and liking was higher for low-caloric relative to high-caloric food. It is likely that these responses were biased by social desirability factors. This hypothesis is supported by the eye-tracking data, which indicated that the first fixation was more often on high-caloric food (than on low-caloric food). This finding is backed by several previous investigations that have also shown that more initial attention (first fixations) is typically directed toward images with high-fat food vs. low-fat food (4, 6). Thus, to summarize, in the current study, the visual preference did not match the verbally expressed preference. To avoid fatigue and boredom, we did not obtain ratings for all images. Thus, the reported preference for the subset of pictures might not be representative for the complete picture set. Moreover, by means of the placebo instruction, we tried to specifically alter the food cue reactivity for high-caloric items. In the context of weight control programs, it would certainly be optimal if the reactivity to high-caloric food could be reduced, while low-caloric food reactivity does not need to change or even could be increased. This goal was not achieved. However, general appetite and focused attention changed in the intended direction. Finally, we did not assess eating behavior in the current experiment. This should be implemented in a future investigation.

In conclusion, the current study provides evidence for a reduction of food cue reactivity *via* placebo. The placebo treatment influenced attentional processes (gaze behavior) as well as food wanting and general appetite. Accordingly, placebos could be a helpful additional component for the treatment of overeating.

## Ethics Statement

The study was approved by the ethics committee of the University of Graz.

## Author Contributions

JP and AS designed the study and wrote the manuscript. JP and NJ recruited participants for the study, collected the data, and conducted the statistical analyses.

## Funding

The authors acknowledge the financial support by the University of Graz.

## Conflict of Interest Statement

The authors declare that the research was conducted in the absence of any commercial or financial relationships that could be construed as a potential conflict of interest.
